# Adult Para Testicular Spindle Cell Rhabdomyosarcoma: A Case Report From Pakistan

**DOI:** 10.7759/cureus.50082

**Published:** 2023-12-06

**Authors:** Noman Ali Ghazanfar, Muhammad Shahzad Anwar, Asad Ali Shah, Humayun Saeed, Muhammad Kashif

**Affiliations:** 1 Urology, Services Hospital, Lahore, PAK

**Keywords:** testicular mass, para-testicular tumor, rhabdomyosarcoma (rms), spindle cell rhabdomyosarcoma, scrotal mass

## Abstract

Para testicular or intra-scrotal Rhabdomyosarcomas (RMS) are rare. The spindle cell variant of rhabdomyosarcoma is the least common variant among embryonal subtypes. They are mostly seen in childhood but rarely reported in adults. We present a case of a 56-year-old man who presented with a three-year history of painless left inguinoscrotal swelling, which he initially ignored and misinterpreted as an Inguinal hernia but later sought medical help upon a progressive increase in the size of the swelling. Clinically and radiologically, there was sparing of the left testis and spermatic cord with normal testicular tumor markers and no evidence of lymphadenopathy or metastasis. Surgical exploration was performed with complete excision of the mass, followed by histopathology and immunocytochemistry, confirming the diagnosis of spindle cell rhabdomyosarcoma. Prompt recognition, early diagnosis, and appropriate surgical treatment are the hallmarks of management. Regular and strict follow-up is needed due to the rarity of diagnosis.

## Introduction

Spindle cell rhabdomyosarcoma is an uncommon variant of embryonal rhabdomyosarcomas, and some also report it to be a different entity among rhabdomyosarcoma subtypes. Among rhabdomyosarcomas, less than 10% are of para-testicular origin [1]. Occurrence of spindle cell rhabdomyosarcomas (RMS) in the para-testicular region, when seen, is mostly in childhood, but rarely it can also be encountered in adulthood [2]. Spindle Cell rhabdomyosarcomas are six times more prevalent in males than females [3]. The diagnostic work-up includes an initial scrotal ultrasound, followed by a chest, abdomen, and pelvis CT for confirmation of findings of scrotal ultrasound as well as for detection of retroperitoneal lymphadenopathy and metastasis. To rule out testicular carcinomas, the basic workup for these para-testicular masses will include conventional testicular tumor markers, i.e., serum Lactate Dehydrogenase (LDH), Serum Alpha-fetoprotein levels, and serum beta hCG levels [4]. There is still a role of fine needle aspiration cytology (FNAC) needle biopsy in case of unclear diagnosis after ruling out testicular carcinomas [5].

In para-testicular spindle cell RMS (Rhabdomyosarcomas), early surgical intervention can be conveniently achieved due to the superficial location of the scrotal mass and is also a critical step in the management of these tumors. The presence of retroperitoneal lymph nodes is a worse prognostic factor that can be adequately assessed radiologically through a CT scan or MRI. Surgical exploration of the retroperitoneum is only reserved for patients with enlarged retroperitoneal lymph nodes on CT scans or MRI [6]. Therefore, complete surgical excision through the inguinoscrotal incision is good enough in the absence of lymphadenopathy on the CT scan. There is no need for surgical exploration of retro-peritoneum for lymphadenectomy [7]. This should be followed by histopathology and immunocytochemistry, which will be pathognomonic.

There is a need to report such rare cases to establish a guideline for management. Strict follow-up and monitoring with urology and oncology are mandated due to the rarity of diagnosis.

## Case presentation

**Figure 1:**
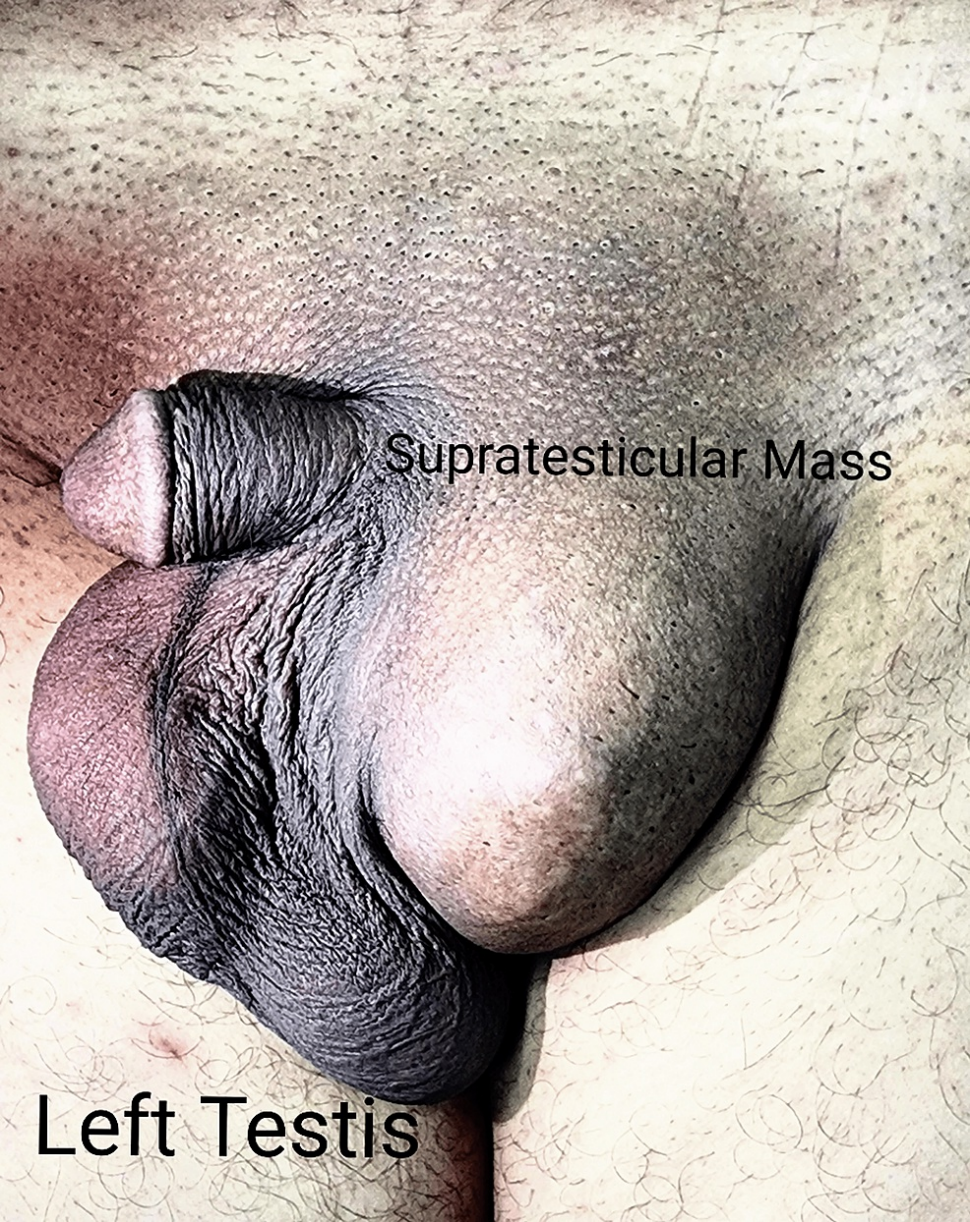
Para-Testicular Mass with Sparing of Testis and Cord

A 56-year-old man presented to us in the Urology outpatient department of Services Hospital Lahore in August 2023 with a three-year history of left painless inguinoscrotal swelling, which was progressively increasing in size. Initially, the patient ignored the swelling as he misinterpreted it as a left inguinal hernia but sought medical help when it started increasing in size. Upon examination, there was a 5x5 cm globular swelling in the left inguinoscrotal region, separately palpable from the left testis and spermatic cord. The swelling was mobile in both planes, non-tender, with no cough impulse, and the overlying skin was mobile-no clinically palpable inguinal or para-aortic lymph nodes (Figure 1). 

He underwent an inguinoscrotal ultrasound, which showed a heterogeneous enlarged mass of about 8x6 cm with loss of normal architecture, showing multiple foci of internal necrosis and significant internal vascularity with arterial blood supply having a resistive index (RI) of 0.5. The contralateral right side was essentially normal. He also had his testicular tumor markers done, including serum alpha-fetoprotein levels, serum LDH levels, and serum beta-hCG levels, which came out to be in the normal range for an adult male (Table 1).

**Table 1:** Blood investigations, including testicular tumor markers INR: international normalised ratio; LDH: Lactate Dehydrogenase; TLC: Total Leukocytes Count; HbA1c: Glycosylated Hemoglobin

S. No	Blood Investigations	Result	Normal Range
1	Serum Alpha-Fetoprotein Levels (α- Fetoprotein)	1.2 ng/ml	(< 5) ng/ml
2	Serum Beta hCG levels (β-hCG)	2 IU/L	( < 2 ) IU/L
3	Serum Lactate Dehydrogenase levels (LDH)	132 U/L	( 135- 225 ) U/L
4	Total Leukocytes Count (TLC)	5.6 /µL	( 4 – 11)/µL
5	Hemoglobin Levels (Hb)	12.2 g/dl	12- 17 g/dl
6	Glycosylated Hemoglobin (HbA1c)	5.90%	< 5.5 %
7	INR (Clotting Profile)	1.1	1- 1.2
9	Serum Creatinine	0.7 mg/dl	0.3 to 1 mg/dl
10	Serum Bilirubin	0.5 mg/dl	0.2 to 1 mg/dl

**Figure 2:**
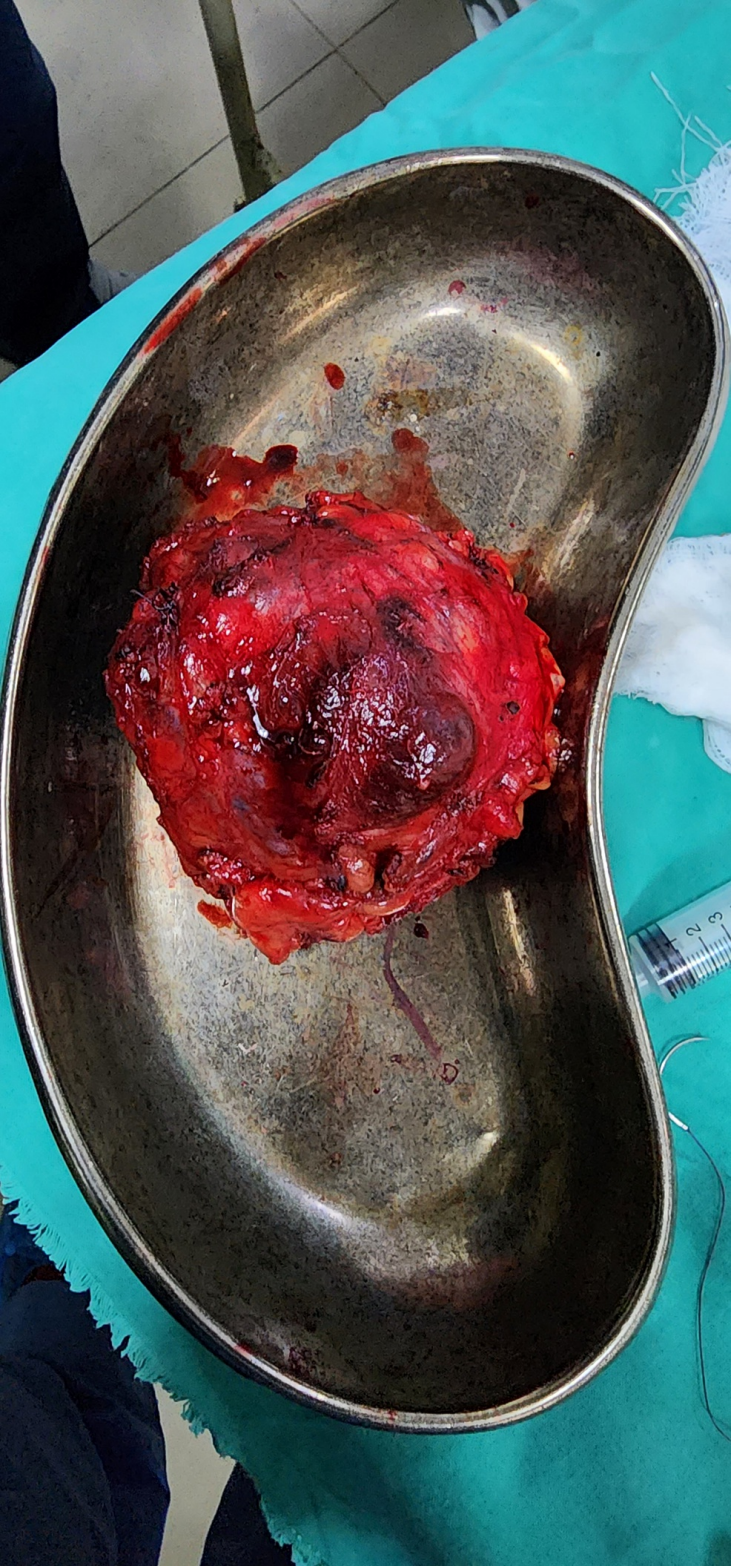
Gross Appearance of Para testicular mass excised

Afterward, the patient underwent a CT scan of the chest, abdomen, and pelvis with contrast, which showed an 8x7x7 cm heterogeneous mass occupying the left scrotal sac with the central non-enhancing necrotic area, posteriorly approaching the medial muscle of the upper thigh with clear fat planes between the two. There was no para-aortic, retroperitoneal, or pelvic lymphadenopathy or metastasis. Due to an unclear diagnosis, the patient had an FNAC needle biopsy of the mass, showing spindle cells and raising suspicion of the presence of para-testicular spindle cell rhabdomyosarcoma. Based upon the above work-up, the patient underwent inguinal exploration under spinal anesthesia with the consent of left orchiectomy. Mass was completely excised with testis and spermatic cord sparing and sent for histopathology and immunocytochemistry (Figure 2).

**Figure 3:**
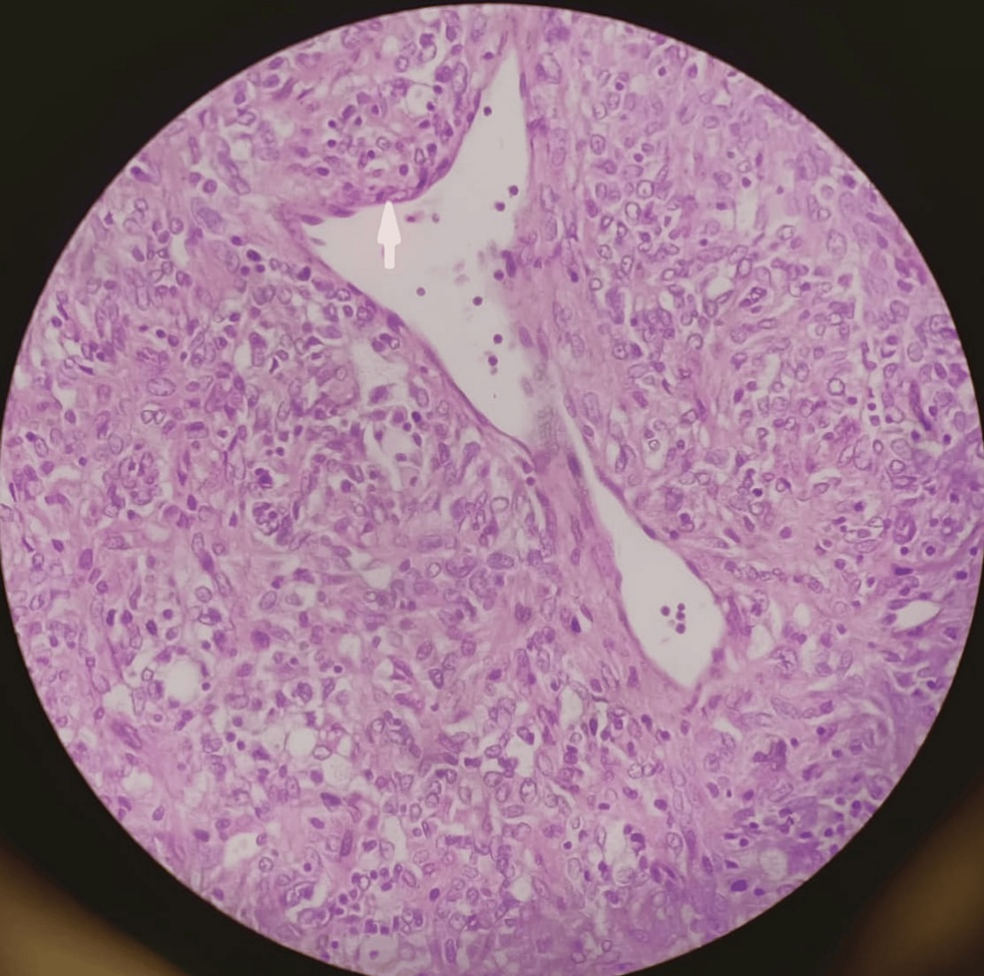
Histopathology of Specimen showing spindle cell neoplasm

Histological examination revealed moderate to highly cellular spindle cell neoplasm composed of sheets of cells having abundant eosinophilic cytoplasm with round to oval nuclei showing moderate nuclear atypia. Few mitotic figures were also seen (Figure 3).

In Immunostaining STAT-6, CD-34 was positive, whereas cytokeratin, SOX-10, TLE-1, and CD-99 were all negative, confirming the diagnosis of spindle cell rhabdomyosarcoma. This was followed by a second review of slides and blocks from an independent source due to the rarity of the diagnosis, reaffirming the same diagnosis.

The patient had an uneventful postoperative recovery and was discharged on the second postoperative day. He remains in strict follow-up of Urology and Oncology due to the rarity of diagnosis.

## Discussion

With para-testicular Spindle cell rhabdomyosarcomas being fairly uncommon, our case was particularly rare due to the presence of such an entity in a 56-year-old man well into his adulthood rather than the common presentation of spindle cell variants in childhood. Worldwide, similar cases have been reported in literature from India, Japan, Turkey, and Syria, but they are far fewer than much more common scrotal or testicular malignancies [8,9]. The use of scrotal ultrasound, CT scan, testicular tumor markers, and FNAC needle biopsy will narrow down the diagnosis. Surgical excision through inguinoscrotal incision will be the treatment of choice [10]. 

There are rarely any cases of such nature being reported from Pakistan, according to our literature search, and our case might be one of the few early reported cases from Pakistan, if not the first. There is a need to gather and report more such cases of Adult para-testicular spindle cell rhabdomyosarcomas to establish guidelines and better understand the natural history of the disease. This will be critical and beneficial in managing such cases, complying with safe surgical practices and patient safety.

## Conclusions

Para-testicular Spindle cell rhabdomyosarcomas in adults is a rare case finding that poses a diagnostic and therapeutic challenge. Clinical presentation may be a superficial scrotal or inguinoscrotal mass with work-up including scrotal ultrasound, CT chest abdomen and pelvis, and FNAC needle biopsy. Surgical excision without retroperitoneal lymphadenopathy is therapeutic, with histopathology and immunocytochemistry being pathognomonic. Strict follow-up is mandated due to the rarity of diagnosis.
